# Variation in early-life telomere dynamics in a long-lived bird: links to environmental conditions and survival

**DOI:** 10.1242/jeb.104265

**Published:** 2015-03

**Authors:** Hannah Watson, Mark Bolton, Pat Monaghan

**Affiliations:** 1 Institute of Biodiversity, Animal Health and Comparative Medicine, University of Glasgow, Glasgow G12 8QQ, UK; 2 RSPB Centre for Conservation Science, UK Headquarters, The Lodge, Sandy, Bedfordshire SG19 2DL, UK

**Keywords:** Telomere length, Life-history evolution, Senescence, Telomere dynamics, Cohort effects

## Abstract

Conditions experienced during early life can have profound consequences for both short- and long-term fitness. Variation in the natal environment has been shown to influence survival and reproductive performance of entire cohorts in wild vertebrate populations. Telomere dynamics potentially provide a link between the early environment and long-term fitness outcomes, yet we know little about how the environment can influence telomere dynamics in early life. We found that environmental conditions during growth have an important influence on early-life telomere length (TL) and attrition in nestlings of a long-lived bird, the European storm petrel *Hydrobates pelagicus*. Nestlings reared under unfavourable environmental conditions experienced significantly greater telomere loss during postnatal development compared with nestlings reared under more favourable natal conditions, which displayed a negligible change in TL. There was, however, no significant difference in pre-fledging TL between cohorts. The results suggest that early-life telomere dynamics could contribute to the marked differences in life-history traits that can arise among cohorts reared under different environmental conditions. Early-life TL was also found to be a significant predictor of survival during the nestling phase, providing further evidence for a link between variation in TL and individual fitness. To what extent the relationship between early-life TL and mortality during the nestling phase is a consequence of genetic, parental and environmental factors is currently unknown, but it is an interesting area for future research. Accelerated telomere attrition under unfavourable conditions, as observed in this study, might play a role in mediating the effects of the early-life environment on later-life performance.

## Abstract (precis)

**Summary:** The environment strongly influences early-life telomere attrition, suggesting that telomere dynamics may underlie cohort effects often observed in natural populations and may mechanistically link early-life stress and later-life performance.

## INTRODUCTION

One of the principal aims of evolutionary ecology is to understand the mechanisms underlying individual variation in longevity and fecundity. Telomere dynamics link cellular processes with organismal ageing and thus optimisation of telomere length (TL) and attrition may play a major role in life-history evolution (Monaghan and Haussmann, 2006). Telomeres comprise highly conserved non-coding DNA sequences that form protective caps at the ends of eukaryotic chromosomes (Blackburn, 2005). By protecting coding sequences from attrition, telomeres play an important role in maintaining genome stability (reviewed by Verdun and Karlseder, 2007). In the absence of the enzyme telomerase, telomeres shorten with each round of somatic cell division; when a critical length is reached, telomeres become dysfunctional and cells enter a state of replicative senescence (Blackburn, 2005; Verdun and Karlseder, 2007). Senescent cells subsequently die or adopt an altered secretory profile, resulting in secretion of inflammatory cytokines, growth factors and degradative enzymes that contribute to age-related declines in tissue and organ function (Campisi, 2005). The accumulation of senescent cells thus appears to be important to the ageing phenotype, thereby influencing lifespan (Campisi, 2005).

Longitudinal studies have shown that TL declines progressively with age in many vertebrates (e.g. Zeichner et al., 1999; Brümmendorf and Mak, 2002; Salomons et al., 2009; Aviv et al., 2009; Bize et al., 2009; Heidinger et al., 2012; Barrett et al., 2013). Large within-species variability in TL and the rate of telomere shortening is reported among individuals of the same age (Hall et al., 2004; Aviv et al., 2009; Bize et al., 2009). Inter-individual variation in TL has been found to predict fitness components in natural populations; individuals with the shortest telomeres or the highest loss rate have the poorest survival prospects (Cawthon et al., 2003; Haussmann et al., 2005; Bize et al., 2009; Olsson et al., 2011; Heidinger et al., 2012) and TL was found to be positively correlated with lifetime reproductive success (Pauliny et al., 2006). Although early-life TL is partly determined by genetic factors (Njajou et al., 2007; Olsson et al., 2011), much of the inter-individual variation in TL may relate to environmental influences. Exposure to repeated stress (Epel et al., 2004; Kotrschal et al., 2007; Herborn et al., 2014), large-scale climatic processes (Mizutani et al., 2013), low habitat quality (Angelier and Vleck, 2013) and reproduction (Kotrschal et al., 2007; Heidinger et al., 2012) have all been associated with accelerated telomere attrition, possibly mediated by increased oxidative damage (von Zglinicki, 2002). TL therefore potentially reflects variation in individual state and past experiences.

**List of abbreviations**

AUC: area under the curve
*C*_t_: the number of PCR cycles required for accumulation of sufficient products to exceed a set threshold of fluorescent signal
GLM: generalised linear model
LM: linear model
LMM: linear mixed-effects model
LRT: likelihood ratio test
OCD: a non-variable copy control gene isolated from the European storm petrel encoding the protein ornithine decarboxylase
qPCR: quantitative PCR
REML: restricted maximum likelihood estimation
*T*/*S*: ratio of telomeric sequence to that of a single-copy gene, relative to the reference sample
TL: telomere length
Δ age: change in age (days) between first and second telomere length measurements

It is well known that early-life conditions can have profound influences on phenotypic development and long-term fitness consequences (Lindström, 1999; Metcalfe and Monaghan, 2001). Variation in the pre- and early post-natal environment has been shown to influence survival and reproductive performance in a number of vertebrate species (Albon et al., 1987; Haywood and Perrins, 1992; Sedinger et al., 1995; Rose et al., 1998; Reid et al., 2003). Environmental conditions during early life can affect an entire cohort simultaneously, giving rise to substantial differences in life histories between successive cohorts, which can persist throughout the cohort's lifespan (Albon et al., 1987; Rose et al., 1998; Reid et al., 2003). Several studies on mammalian and avian species have found that telomere loss is greatest during early life, presumably as a result of the rapid growth and cell division that occurs during this period (Zeichner et al., 1999; Baerlocher et al., 2007; Salomons et al., 2009). Poor early nutrition and catch-up growth have been shown to result in accelerated telomere loss in a number of studies, including laboratory rats (*Rattus norvegicus*: Jennings et al., 1999; Tarry-Adkins et al., 2009) and wild birds (European shag *Phalacrocorax aristotelis*: Hall et al., 2004; king penguin *Aptenodytes patagonicus*: Geiger et al., 2012). Developmental stress has also been shown to accelerate telomere shortening through experimental brood enlargement (jackdaw *Corvus monedula*: Boonekamp et al., 2014) and increased exposure of nestlings to glucocorticoids (European shag: Herborn et al., 2014) in the wild. Early-life TL was found to be a better predictor of longevity than TL in adulthood in a longitudinal study of captive zebra finches *Taeniopygia guttata* (Heidinger et al., 2012). Early-life telomere dynamics may therefore mechanistically link developmental conditions with later-life senescence (Monaghan, 2010). Despite the potential significance of early-life telomere dynamics in influencing life-history traits, we still know relatively little about how variation in early-life conditions influences telomere loss during development in natural environments.

In this study, we examined the effects of inter-annual variation in the natal environment on TL and telomere dynamics during postnatal development in the European storm petrel *Hydrobates pelagicus* Linnaeus 1758 (hereafter, storm petrel). The storm petrel is a remarkably long-lived seabird, displaying low annual fecundity (obligate clutch of one) and low adult mortality. Its long lifespan may in part be attributed to superior cellular mechanisms for resistance to oxidative damage (Ogburn et al., 2001) and maintenance of telomere length (Haussmann et al., 2007). The oldest individuals in a population of a related species, the Leach's storm petrel *Oceanodroma leucorhoa*, show little or no accumulation of short telomeres over time (Haussmann and Mauck, 2008). We compared cohorts from two consecutive breeding seasons characterised by different environmental conditions, as indicated by overall reproductive performance of the colony. Productivity was relatively poor in 2011 and significantly lower than 2010 (Watson et al., 2014). This provided the opportunity to examine the effects of the natal environment on telomere length and dynamics during early life and relate these to nestling survival. The study site and design also enabled us to investigate whether exposure to an additional source of developmental stress, arising from human recreational disturbance, has consequences for early-life telomere dynamics. Parent–offspring relationships in TL were examined within a single cohort.

## RESULTS

**Fig. 1.**
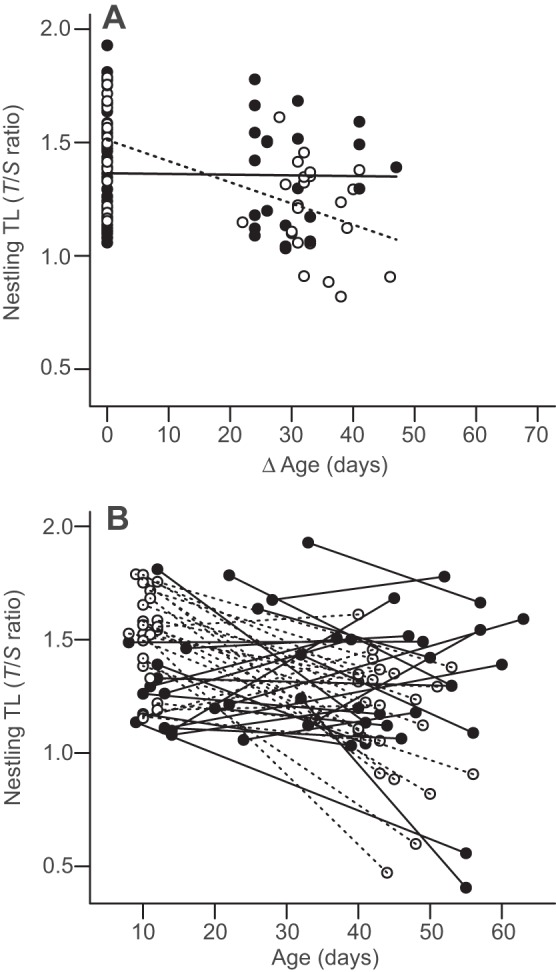
**Change in telomere length during growth in storm petrel nestlings from two consecutive cohorts reared under different natal conditions.** (A) Mean within-individual change in TL in 2010 (black circles; solid line) and 2011 (open circles; dashed line). Lines represent model predictions from the minimum adequate LMM (Δ age×cohort (2011): *F*_1,55.2_*=*12.17, *P*≤0.001, *N*=100) fitted within the range of observed values. Δ age is the change in age (days) between the first and second measurement. (B) Individual change in telomere length during postnatal development (*N=*98) in 2010 (black circles; solid lines) and 2011 (open circles; dashed lines). Lines link TL measurements in early and late postnatal development for each individual.

The within-individual change in nestling TL was significantly different between the two cohorts raised under different environmental conditions: in 2010, there was little change in TL during the nestling period, whereas TL declined significantly with age in nestlings reared in 2011 (Table 1, model 1; Fig. 1A; Δ age×cohort: *F*_1,55.2_*=*12.17, *P*≤0.001). However, TL in early postnatal development was significantly longer in individuals from the 2011 cohort, compared with the 2010 cohort (Table 1, model 1; *F*_1,94.3_*=*6.25, *P=*0.014). Removal of the main effect of cohort, while retaining it in the interaction with Δ age (change in age in days between first and second telomere length measurements), showed that, even after adjusting for early differences in TL, the rate of change in TL was still significantly different between the two cohorts (β=−0.005±0.002, *F*_1,95.5_*=*5.82, *P*=0.018) and therefore the cohort effect was above and beyond any effect due to regression to the mean. Furthermore, there was considerable variation between individuals in the degree of change in TL, with some individuals even exhibiting an apparent increase in TL during postnatal development (Fig. 1B). The between-individual differences in TL were not quite significant (Table 1, model 1; age at first measurement: *F*_1,72.6_=3.81, *P*=0.055). Neither nestling TL nor the within-individual change in TL (tested by the respective interactions with Δ age) was affected by any of the other variables considered, including sex, hatching date or visitor disturbance (all *P*>0.2). Analysis of TL in late postnatal development demonstrated that, although there was a tendency for late TL to be shorter in 2011, the estimated cohort effect was not quite significant at this stage (Table 1, model 2; *t*_47_*=*−1.73, *P=*0.089).

**Table 1.**
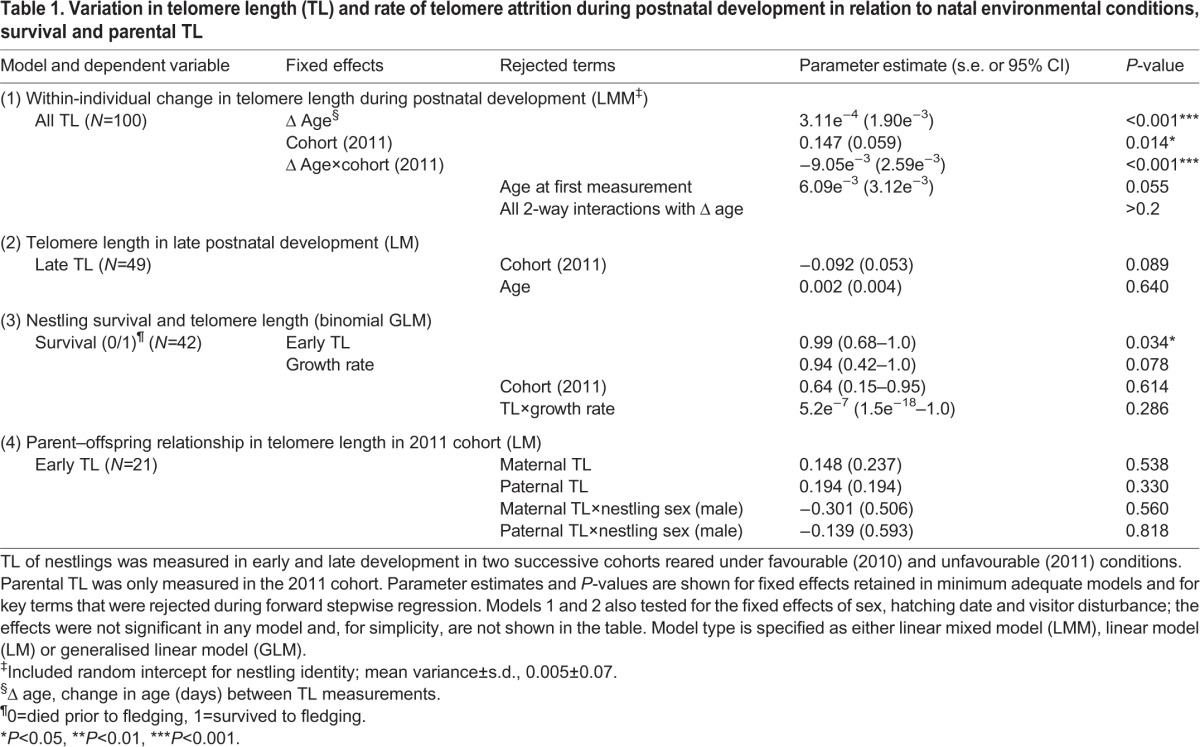
**Variation in telomere length (TL) and rate of telomere attrition during postnatal development in relation to natal environmental conditions, survival and parental TL**

**Fig. 2.**
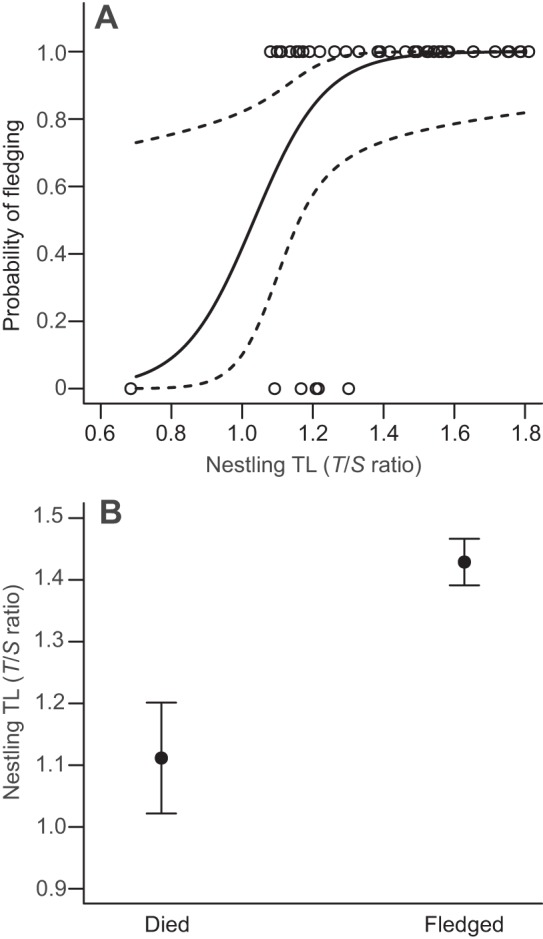
**Relationship between early nestling telomere length and survival during postnatal development.** (A) Predicted probability of fledging (solid line) in relation to early TL (≤16 days; GLM: *z*_39_=2.12, *P*=0.034) with 95% confidence intervals (dashed lines). Line is fitted within the range of observed values (circles; *N=*42) and based on a mean rate of mass gain of 1.03 g day^−1^ (*z*_39_=1.76, *P*=0.078). Removal of the outlier at the lower end of the TL scale did not affect model predictions. (B) Mean±s.e. early TL of nestlings that died (*N=*6) during the nestling phase and those that survived to fledging (*N=*36).

Of the 61 nestlings sampled, 6 died prior to fledging (3 individuals from each cohort). When examining whether early TL was a good predictor of survival of the nestling phase, we found that the probability of surviving to fledging increased significantly with TL (Table 1, model 3; Fig. 2A; *z*_39_=2.12, *P=*0.034). The mean TL of chicks that did not survive to fledging was 22% shorter than that of chicks that successfully fledged (Fig. 2B). The model fit was significantly improved by the inclusion of the rate of mass gain, though this variable did not explain a significant portion of the variation in probability of fledging (Table 1, model 3; *z*_39_=1.76, *P=*0.078). One of the chicks that did not fledge had very short telomeres (*T*/*S* ratio=0.68) and so the analysis was re-run excluding this nestling. The outlier had no influence on the relationship, its removal having no effect on the magnitude or significance of the effect of TL (β*=*0.99, 95% CI: 0.64–1.0, *z*_38_=2.08, *P=*0.038). The probability of fledging was not affected by sex, hatching date, cohort or visitor disturbance (all *P*>0.1). The relationship between nestling TL and probability of fledging was not affected by rate of mass growth (Table 1, model 3; TL×growth rate: *z*_38_= −1.07, *P*=0.286). The AUC of the minimum adequate model was 0.90, indicating that the model performed very well in terms of accuracy.

Analysis of parent–offspring relationships in the 2011 cohort revealed that nestling TL was not significantly correlated with either maternal TL (Table 1, model 4; *t*_19_*=*0.63, *P=*0.538) or paternal TL (Table 1, model 4; *t*_19_*=*1.0, *P=*0.330). Parent–offspring TL relationships were unaffected by nestling sex, as shown by testing the respective two-way interactions (all *P*>0.5).

## DISCUSSION

Although it has been widely suggested that the most rapid telomere loss occurs early in life in a range of vertebrates (Zeichner et al., 1999; Baerlocher et al., 2007; Salomons et al., 2009), only a few studies have investigated telomere dynamics within the timeframe of postnatal development itself (see Foote et al., 2011; Geiger et al., 2012; Boonekamp et al., 2014; Herborn et al., 2014). We found that TL and dynamics in growing nestlings of a long-lived species were strongly influenced by the quality of the natal environment. Favourable environmental conditions during development, as reflected by moderate colony productivity in 2010, were associated with a negligible change in TL during early life, whereas conditions resulting in significantly lower chick survival among the 2011 cohort were associated with significant telomere attrition. Whereas human recreational disturbance was previously shown to reduce survival probabilities of nestlings (Watson et al., 2014), there was no effect of visitor activity on telomere length or dynamics.

While some of the cohort effect might be explained by regression to the mean, the difference in rate of change in TL remained, even after adjusting for the differences in early TL between the two cohorts. Accelerated attrition of longer telomeres, having controlled for regression to the mean, has previously been reported (Salomons et al., 2009; Aviv et al., 2009) and could be explained if longer telomeres offer a larger area for attack by free radicals (Aviv et al., 2009). The high early TL in the 2011 cohort could potentially be explained if the high early mortality observed within the 2011 cohort removed individuals with short telomeres from the population or if poor-quality and/or inexperienced birds did not breed. Early selective mortality of individuals with short telomeres was previously predicted to occur in storm petrels (Haussmann and Mauck, 2008). Environmental conditions could be driving the accelerated telomere attrition observed in the 2011 cohort either directly, for example, via elevated oxidative stress, and/or indirectly, if selective mortality or breeding by only high-quality individuals were to result in a higher mean early TL, which is then subject to increased attrition. The functional links between the environment and telomere dynamics could be mediated by changes in parental care. Using a larger dataset from the same population, we found that, in 2011, parents spent less time brooding their young and maximal growth rate (in respect of mass) occurred later (H.W., unpublished results). An increased frequency of extreme weather events in 2011 (Watson, 2014) might have been a key factor influencing breeding success and parental care. Irrespective of the mechanism(s), this study presents good evidence for environmental conditions influencing the rate of telomere loss and shaping variation in early-life telomere dynamics between cohorts.

It is well known that cohort effects can arise in response to natal conditions. Differences in survival and recruitment rates among cohorts have previously been shown to be positively correlated with the natal environment in mammals (Albon et al., 1987; Rose et al., 1998) and birds (Sedinger et al., 1995; Reid et al., 2003). An increasing body of evidence links short telomeres and accelerated rate of telomere loss to a shorter lifespan (Cawthon et al., 2003; Bize et al., 2009; Olsson et al., 2011; Heidinger et al., 2012) and lower reproductive success (Pauliny et al., 2006). The results of this study suggest that early-life telomere dynamics may contribute to the marked differences in life-history traits that can arise among cohorts reared under different environmental conditions. Indeed, TL in early life has been shown to be a strong predictor of lifespan and a better predictor than TL in adulthood (Heidinger et al., 2012). In addition to the increase in chick mortality observed in the cohort reared under unfavourable natal conditions, the population-level effects might be exacerbated by a reduction in the fitness of entire cohorts compared with those fledging under more favourable conditions. Such cohort-wide effects can subsequently destabilise population dynamics (Lindström, 1999). Although our understanding will benefit from examination of longer-term data of multiple cohorts, this study supports the idea that telomere dynamics may be a mechanism linking early-life conditions with later-life performance.

The maintenance of TL during postnatal development under favourable conditions in storm petrels contrasts with studies in other birds that suggest rapid telomere loss invariably occurs early in life (Hall et al., 2004; Baerlocher et al., 2007; Salomons et al., 2009; Heidinger et al., 2012). A negligible change in early-life TL was also demonstrated in chicks of the long-lived king penguin experiencing favourable growth conditions (Geiger et al., 2012). A slow rate of telomere shortening could be causally linked to a higher resistance to oxidative stress (Ogburn et al., 2001) and/or elevated telomerase activity (Verdun and Karlseder, 2007; Haussmann et al., 2007). Stressful conditions, however, have been shown to lead to accelerated telomere loss via downregulation of antioxidants in birds (Stier et al., 2009) and telomerase in humans (Epel et al., 2004). When faced with a costly reproductive event, adult Adélie penguins *Pygoscelis adeliae* are able to increase antioxidant defences and avoid accelerated telomere attrition (Beaulieu et al., 2011). The results of this study suggest that storm petrel nestlings, when faced with unfavourable natal conditions, are unable to modulate or activate such regulatory mechanisms. If poor early conditions were to lead to irreversible downregulation of antioxidants or telomerase, accelerated telomere loss may even persist beyond the nestling period, exacerbating the effects of early conditions on later-life senescence. An area for future research is to understand the links between telomere loss, oxidative stress and telomerase activity during early life.

While recent studies have investigated the relationship between TL in early life and adult survival in captive (Heidinger et al., 2012) and wild (Caprioli et al., 2013; Boonekamp et al., 2014) birds, we are aware of only one study that has directly examined the association between early-life TL and mortality within the development phase itself. Just as king penguin chicks that died prior to fledging displayed shorter telomeres (Geiger et al., 2012), we also found that post-hatching TL of storm petrel nestlings was directly related to imminent mortality. Despite a small sample size, the model performed well (as measured by AUC) and the results were not influenced by an outlying observation. Although confidence intervals are wide at low TLs, they do not overlap with confidence intervals when the probability of fledging is equal to one. TL was a much better predictor of fledging success than the rate of mass gain. Although food load and rate of mass gain are likely to be of importance in determining survival within the first few days of hatching, storm petrel nestlings are well-buffered against periodic food shortages and mortality beyond the brooding stage is unlikely to be strongly influenced by environmental factors affecting rate of provisioning and subsequent mass gain. Additionally, because they nest in cavities, the risk of nest predation is very low. Consequently, it seems most likely that mortality was linked to intrinsic, rather than extrinsic, factors. Accelerated telomere shortening at this stage is likely to be a biomarker rather than a cause of poor survival prospects and may be indicative of exposure to high levels of glucocorticoids and/or oxidative stress. Early TL is partly determined by genetic factors (Njajou et al., 2007), but the rate of attrition is also known to be affected by environmental influences (Epel et al., 2004; Tarry-Adkins et al., 2009; Geiger et al., 2012). The results demonstrate that early-life TL is a good predictor of imminent mortality.

Recent evidence suggests that TL in birds is maternally inherited (an example of heterogametic inheritance) (Horn et al., 2011; Reichert et al., 2015; Asghar et al., 2015). However, Reichert et al. (2015) also showed that environmental factors exerted a strong influence on nestling TL and the maternal link disappeared with chick age. The absence of any observable heritable component to early TL in this study may therefore be a consequence of environmental effects obscuring any initial genetic effect on nestling TL. As we only have data on parent–offspring relationships for the cohort reared under unfavourable conditions, parent–offspring relationships in this study could have been obscured by parental age, which was not known; although, telomere length was found to be unrelated to age in two other long-lived avian species (Hall et al., 2004). Future research should seek to disentangle the relative contributions from genetic, parental and environmental effects on TL and dynamics. This will help to develop our understanding of the role of telomere dynamics in driving the evolution of life histories.

## MATERIALS AND METHODS

### Data collection

The study was conducted at the island of Mousa, located in the Shetland archipelago, UK (60°0′N, 1°10′W). Storm petrel nestlings are brooded for ∼7 days and do not leave the underground nest cavity until fledging at ∼65–70 days (Davis, 1957). Fledging success (of eggs hatched) was significantly lower in 2011 (0.60), compared with 2010 (0.78) (see Watson et al., 2014). This was probably linked, in part, to an increased frequency of extreme weather events in 2011 (Watson, 2014). It is not always clear what environmental correlates are relevant, however, and overall breeding performance of a population is widely used as a measure of the natal environment (e.g. Reid et al., 2003). Blood was collected from 32 nestlings in 2010 and 29 nestlings in 2011 and from both parents at 21 nests in 2011 only. Whole blood was obtained by venipuncture of the brachial vein under licence from the UK Home Office. Nestlings were sampled on two occasions (where possible) in the first (hereafter, ‘early’; median age: 11 days, range: 4–33 days, *N=*59) and second (hereafter, ‘late’; median: 45 days, range: 34–63 days, *N=*52) half of postnatal development. Fifty nestlings were sampled twice and the median number of days elapsed between repeated measurements was 31 days (range: 22–47 days). Only one sample was available for some nestlings, either because nestlings died (*N=*6), were not accessible in the nest or samples were accidentally destroyed. Of the 50 nestlings sampled twice, 49 survived to fledging. The overall proportion of chicks that died was greatly underrepresented in the data and the difference in mortality between the two cohorts was not reflected within the dataset; this is because the majority of chick mortality occurred within a few days of hatching and before initial blood sampling was carried out. Tarsus length (nestlings only), mass and wing length (maximum flattened chord; adults only) were recorded. Nestling mass was corrected to a standardised time (18:00 h) to account for the age-related rate of proportional weight loss that chicks undergo during diurnal fasting (Bolton, 1995).

### Determination of TL

Blood samples were stored at <5°C for up to 8 h prior to being separated by centrifugation. Plasma and red blood cells were stored at <5°C in the field for a maximum of 3 days before being transferred to −20°C for up to 3 months; following this, samples were stored at −80°C until laboratory analyses were performed. DNA was extracted from red blood cells using Machery–Nagel NucleoSpin Blood kits following the manufacturer's protocol. The quantity and purity of DNA was assessed using a NanoDrop 8000 spectrophotometer (Thermo Scientific); there were no differences in yield or purity between cohorts. Sex was determined following the molecular method described by Griffiths et al. (1998). TL was measured by quantitative PCR (qPCR) using an Mx3005P machine (Stratagene) as described by Cawthon (2002) and adapted by Criscuolo et al. (2009) for birds. The method gives a relative value for TL by determining the ratio of number of telomere repeats (*T*) to that of a single-copy (or, more precisely, non-variable in copy number) control gene (*S*) relative to a reference sample. Consequently, the measure will include any interstitial repeats of the telomeric sequence (Foote et al., 2013). However, good correlations have been found between TL measurements including and excluding the interstitial repeats using telomere restriction fragment (TRF) analysis (Foote et al., 2013) and between TRF and qPCR analyses (Criscuolo et al., 2009; Aviv et al., 2011). Since the extent of interstitial repeats is not expected to change with age, qPCR is well suited to detecting within-individual changes. Furthermore, measurement of telomeres from Leach's storm petrels, using TRF methods, suggests that interstitial repeats do not occur to the extent that they would influence telomere measurements (M. Haussmann, personal communication).

Amplification of telomere sequences was achieved using the forward and reverse primers: Tel1b (5′-CGGTTTGTTTGGGTTTGGGTTTGGGTTT-GGGTTTGGGTT-3′) and Tel2b (5′-GGCTTGCCTTACCCTTACCCTT-ACCCTTACCCTTACCCT-3′). The non-variable copy control gene used was ornithine decarboxylase (OCD), isolated from the European storm petrel (GenBank: DQ881744.1), which was amplified using the primers: OCD Fwd1 (5′-GACCTTGCCATCATTGGAGTTAG-3′) and OCD Rev1 (5′-AAGGCATCCCTATTGTTAGGTAGA-3′) sourced from Integrated DNA Technologies (Leuven, Belgium). qPCR was performed using 10 ng DNA per reaction. The concentrations of primers used were 500 nmol l^−1^ for telomere and 70 nmol l^−1^ for OCD reactions. Telomere qPCR reaction conditions started with 15 min at 95°C, followed by 27 cycles of 15 s at 95°C, 30 s annealing at 58°C and 30 s extending at 72°C. OCD reaction conditions started with 15 min at 95°C, followed by 40 cycles of 30 s at 95°C and 30 s at 60°C. Melting curves were assessed to confirm there was no non-specific amplification or primer–dimer formation. For both telomere and OCD reactions, the number of PCR cycles required for accumulation of sufficient products to exceed a threshold of fluorescent signal (*C*_t_) was determined. A standard curve, run on each plate, consisted of a serial dilution of a reference sample ranging from 40 ng to 2.5 ng. The *C*_t_ threshold for each reaction was determined from the reference sample. A negative control was also included on each plate. All samples, including the standard curve, were run in triplicate. Amplification efficiencies were within an acceptable range (mean±s.d.: telomere, 106.8±3.7%; OCD, 114.3±3.9%) and all samples fell within the bounds of the standard curve. Inter- and intra-assay variations were 3.6% and 1.4%, respectively, for telomere reactions and 0.82% and 0.33%, respectively, for OCD reactions. Inter-assay variation in *T*/*S* ratio was 10.4%. The *T*/*S* ratio for each sample was calculated from mean *C*t values according to Pfaffl (2001). This method accounts for the variation in amplification efficiencies. All repeated samples from nestlings were run on the same plate along with parental samples.

### Statistical analysis

All statistical analyses were performed in R 3.0.0 (R Core Team, 2013). Linear mixed models (LMMs) with a normal error structure were fitted to data on nestling telomere length to examine change in TL in surviving nestlings (*N=*100; number of individuals=55). Outlying observations were removed to normalise the data and improve model fits. Because we were primarily interested in the within-individual change in nestling TL, we used within-subject centring (van de Pol and Verhulst, 2006) to separate between-individual (cross-sectional) and within-individual (longitudinal) effects. The age variable was split into two covariates: age at first measurement (between-individual effects) and Δ age (the change in age between measurements; within-individual effects). LMMs were fitted in the lme4 package (Bates et al., 2013). First, the optimal random effects structure was found by comparing nested models, fitted by restricted maximum likelihood (REML), using likelihood ratio tests (LRTs). A random intercept for nestling identity was included. We also considered the inclusion of a random intercept for nest identity, to account for potential non-independence of nestlings from different cohorts reared at the same nest-site. Thirty per cent of nests were sampled in both years, although we do not know whether nests were occupied by the same breeding pair in both years. However, the variance associated with nest identity was estimated to be zero and inclusion of the random effect did not significantly improve model fits. With nestling TL as the dependent variable, candidate explanatory variables included cohort (year: 2010 or 2011), nestling sex and human disturbance (high or low, defined as ≤10 m or >150 m from the visitor path, respectively) as two-level fixed factors, and age at first measurement, Δ age and hatching date as covariates. We tested for effects on within-individual change in TL by considering all respective interactions with Δ age. To examine variation in nestling TL in late development, linear models (LMs) with a normal error structure were fitted to log-transformed late nestling TL. Candidate explanatory variables included cohort, nestling sex and human disturbance as fixed factors and age and hatching date as covariates.

Generalised linear models (GLMs) with a binomial error structure and logit link function were fitted to data on survival (0/1; *N*=42) to examine whether early TL (age: ≤16 days, median=11 days) was a good predictor of survival of the nestling phase. The threshold of 16 days was adopted because this was the upper limit of the 95% confidence interval of age at first measurement. These data did not include any repeated measures at the nest level. In addition to the fixed effect of nestling TL at ≤16 days, candidate explanatory variables included cohort, sex and visitor disturbance as fixed factors and body size, body mass, hatching date, growth rate (average daily incremental growth in mass) as covariates. We also tested for two-way interactions between TL and mass growth rate. The minimum adequate model was evaluated using receiver operating characteristic plots (Sing et al., 2005). The resulting area under the curve (AUC) offers a measure of predictive performance for a binomial model; a value of 1.0 indicates a perfect model, whereas a value of 0.5 indicates that a model performs no better than random.

Finally, parent–offspring relationships in the 2011 cohort were examined in a linear model (LM) analysis with early nestling TL as the dependent variable (*N*=21). Candidate fixed effects included the covariates of maternal TL, paternal TL and nestling sex as a fixed factor. We also tested for potential effects of nestling sex on parent–offspring relationships with the respective two-way interactions between parental TL and nestling sex. We showed in previous analyses that age, hatching date and visitor disturbance did not affect early nestling TL and these variables were therefore not included in the parent–offspring analysis.

Taking into account the sample sizes and the number of variables to potentially control for, a conservative approach to model fitting was adopted; starting from a null model, fixed effects that significantly differed from zero (*P*<0.05) were added sequentially in a forward stepwise regression. Each time a new variable was added to the model, the significance of existing variables was re-examined. Nested models were compared using LRTs and the criterion for entry of a variable was a log-likelihood ratio *P-*value of <0.05. For LMMs, model selection was performed using models fitted by maximum likelihood; parameter estimates with standard errors are quoted from the minimum adequate LMM fitted by REML. The significance of parameter estimates was estimated using conditional *F*-tests based on Satterthwaite approximation for the denominator degrees of freedom. For LMs and GLMs, parameter estimates were estimated based on the *t*- and *z*-distributions, respectively; again estimates with standard errors are quoted from minimum adequate models.
